# No Trends in the Age of Peak Performance among the Best Half-Marathoners and Marathoners in the World between 1997–2020

**DOI:** 10.3390/medicina57050409

**Published:** 2021-04-23

**Authors:** Mabliny Thuany, Thayse Natacha Gomes, Thomas Rosemann, Beat Knechtle, Raphael Fabrício de Souza

**Affiliations:** 1 Centre of Research, Education, Innovation and Intervention in Sport (CIFI2D), Faculty of Sports, University of Porto, 4200-450 Porto, Portugal; mablinysantos@gmail.com; 2 Department of Physical Education, Federal University of Sergipe (UFS), São Cristóvão 49100-000, Sergipe, Brazil; thayse_natacha@hotmail.com (T.N.G.); raphaelctba20@hotmail.com (R.F.d.S.); 3 Post-Graduation Program of Physical Education, Federal University of Sergipe (UFS), São Cristóvão 49100-000, Sergipe, Brazil; 4 Institute of Primary Care, University of Zurich, 8091 Zurich, Switzerland; thomas.rosemann@usz.ch; 5 Medbase St. Gallen Am Vadianplatz, Vadianstrasse 26, 9001 St. Gallen, Switzerland

**Keywords:** running, age peak performance, athletes

## Abstract

*Background and Objectives*: We examined the possible trend in the age of peak performance in elite endurance athletes according to sex, continent of athletes’ national citizenship, and ranking position. Since performance is a multifactorial trait, this information can be used to guide the long-term training and to plan the strategies related to the selection process of athletes. *Materials and methods*: Information of 1852 professional athletes, classified as top 20 performance of each year in marathon and half-marathon events between 1997 and 2020 were considered. Analysis of variance was computed to test differences in age between sex, continent, and rank position. *Results:* A significant difference between groups in the mean age of peak performance was observed (F (3, 1884) = 42,31; *p* < 0.001). For both sexes, half-marathoners were younger than marathoners (male, 25.6 ± 3.6 years vs. 28.0 ± 3.9 years; female, 27.5 ± 4.4 years vs. 28.4 ± 4.1). Female half-marathoners in 2004 presented the highest mean age (31.1 ± 4.8 years) compared to their peers in the years 1997, 2001, 2018 and 2019; among male half-marathoners, those in 1999 presented the highest mean age when compared to 2011, 2018, and 2019. Differences between the continents of athletes’ national citizenship were observed (F (4, 1884) = 62,85,601; *p* < 0,001). Asian runners presented the lowest mean age (26.5 ± 3.7 years), while their European peers presented the highest (31.1 ± 3.9 years). No significant interaction between sex and ranking position was verified. Differences were observed between sexes for categories “4th–10th positions” and “11th–20th” (F (1, 1879) = 23,114; *p* < 0.001). *Conclusions:* Over the last two decades, no clear trend was observed in the changes in the age of peak performance among endurance athletes of both sexes, but, in general, female half-marathoners tended to be significantly older than their male peers.

## 1. Introduction

The peak of athletic performance is usually reached, in most sports, during early adulthood and decreases thereafter [1]. Since performance is a multifactorial trait developed during the lifespan [2], knowing the age of peak performance in a given sport may be relevant for athletes and coaches to guide the long-term training and plan the strategies related to the selection process of athletes [3].

Since the 1970s, an increase in the popularity of long-distance events such as half-marathon and marathon races has been observed. This may be related to the fact that running is considered accessible for subjects of different ages and economic conditions and its challenging characteristics [4]. Although maximum oxygen uptake (VO_2max_), pointed as one of the best physiological predictors of performance in endurance sports, decreases by ≈1% per year after 25 years, peak performance among professional marathon runners seems to present a U-shaped curve [5]. This means that despite a decrease in performance observed after the peak reached in early years (usually at 20 s), it is possible to observe a further increase in performance in late ages, with a possible second peak that can be reached around 30 years. For example, the non-official status of this record notwithstanding [6], the world was able to see a remarkable feat performed by a 35-year-old marathoner (Eliud Kipchoge, in Vienna, in 2019, who was 8 years older than the half-marathon world record holder in the same year), who covered the marathon distance in under two hours [7]. In addition, a case study conducted with a world female distance runner during three consecutive years identified that the improvement in 3000 m running performance was not caused by an increase in VO_2max_ but was related to both running economy and lactate threshold, which considerably improved during the follow-up [8].

In general, it is observed that endurance sports athletes, such as those competing in track and field [9], middle and long-distance running [10], and ski marathon [11], seemed to reach their peak performance in older ages when compared to athletes from sports disciplines requiring explosive power and speed [3]. In addition, some sex differences in the age of peak performance in runners have also been pointed out. For example, data from the Boston, Chicago, and New York marathons between 2001 and 2016 indicated that elite women tended to achieve their peak performance in these races about 1 ± 2 years later than their male peers [5]. Data indicated that the mean age for the overall champions was 27.3–29.4 years for men and 29.9–31.8 years for women. Another aspect observed concerns age-related performance decline: the highest percentage of decline in the winners finishing times was associated with aging and not sex. Some researchers have suggested a slight change in peak age performance among female runners, leading it closer to male’s [12].

Furthermore, another aspect that should be observed regarding the age of peak performance is related to given individual factors (e.g., physiological and anthropometric characteristics, training), which are usually associated with the performance in running [13], its expression also varies according to runners’ country of citizenship. In this context, the dominance of African endurance runners at world-class levels was previously described [14,15], and predictors pointed as possible related to this factor the demographic and genetic characteristics, as well as environmental factors, high running economy, and sociocultural background [15,16,17,18].

Regarding the age of peak performance in African runners, Knechtle et al. [16], studying 508,180 half-marathoners and marathoners from 126 countries, identified that Ethiopian and Kenyan runners, from both sexes, achieved their best marks in youngest ages than their peers (male: non-African 31.1 ± 6.4, African runners 26.2 ± 4.9; female: non-African runners 33.0 ± 4.8, African runners 27.8 ± 5.3). Moreover, when the age of peak performance among marathoners was investigated based on four of the Marathon Majors and the Stockholm Marathon (2000–2014), a similar scenario was found. Ethiopian athletes were the youngest (27.3 ± 5.6 years to men; woman: 28.1 ± 4.8) among the best runners (highest value was observed to Morocco 37.4 ± 6.7 and Austria 38.9 ± 6.9 to men and woman, respectively) [19]. These results can be related to the fact that economic reasons are pointed as one of the most relevant motivation factors to African runners get engaged into the practice [18], which also contributes to their earlier specialization in their careers [20], which can help them to reach their peak performance earlier in life than their peers from other nationalities. Given that, we hypothesized that African runners were younger than their peers from other continents.

Additionally, since endurance performance is mainly determined by aerobic capacity, running economy, and lactate threshold [21], which are developed by training/practice and also improved with the time spent in practice [7], and given marathon runners tended to present a higher aerobic resistance and lower muscle power, we hypothesized that marathon athletes were older than half-marathoners. Furthermore, the increase in women participation in running events, in association with changes in society’s beliefs and patterns regarding opportunities for women engagement in sports in the last decades, which allowed them to compete until late ages, we believed that a more prominent trend in changes of peak performance could be observed among female compared to male runners. Based on this scenario, we also hypothesized that the peak age performance of worldwide best marathon and half-marathon runners has changed throughout the last two decades.

Therefore, the purposes of this study were (1) to identify the trend in the peak age performance in endurance athletes for both sexes of marathon and half-marathon between 1997 and 2020 and (2) to identify differences in the peak age performance between continents and ranking positions (top 3-ranking positions; between the 4th and 10th ranking positions; and others—from the 11^th^ to 20^th^ ranking positions).

## 2. Materials and Methods

### 2.1. Sample and Design

This is an exploratory study using information obtained from official publications. Data were collected from the results section of the Tilastopaja website (www.tilastopaja.eu/) during November 2020. Information refers to results available for the athletes presented in the top-20 worldwide ranking best half-marathon and marathon marks in official outdoor events, comprising the period between 1997 and 2020 for both sexes. The sample comprised 1932 professional athlete runners (963 female and 969 male), aged 18–41 years, who were ranked in the senior world’s top 20 half-marathon (483 female and 487 male) and marathon races (480 female and 482 male) between the years 1997 and 2020. Information used included: athlete’s name, date of birth, sex, race time, national citizenship (which was used to identify the continent where the country of citizenship belongs to), date of the competition, and athlete position in the worldwide ranking in the modality in the year (taking into account the best marks of all runners, for a given race, in official events). The athlete’s age was computed, taking into account the date of birth and the date of the competition, and we considered this information to determine the age of peak performance. In addition, based on ranking position, athletes were categorized as “until the 3rd position”, “4th–10th positions,” and “above the 10th position.

### 2.2. Statistical Analysis

Descriptive information was expressed as mean (and standard deviation) and median (and interquartile range) or frequency (%). Data normality distribution was tested by the Kolmogorov–Smirnov test, split by sex. One-way ANOVA was performed to test mean age differences between the continent of athletes’ national citizenship, and further to test for differences among sex, with the Bonferroni post hoc test. To observe age differences during 1997–2020, one-way ANOVA was performed for sex and running distance, followed by Bonferroni post hoc test (differences (Δ) in age mean were presented). Further, taking into account athletes categories based on their ranking position (“until the 3rd position”, “4th–10th positions” and “11th–20th”), a two-way ANOVA was performed to identify mean age differences between ranking position and sexes, with a post hoc test (Bonferroni), used when necessary. SPSS 26.0 software was used in all analyses, and the significance level was set at 0.05.

## 3. Results

The sample comprised athletes from both sexes (49.8% women; 50.2% men) with a mean age of 27.4 ± 4.19 years. Taking into account sex and race distance, a significant difference between groups in the mean age of peak performance was observed (F (3, 1884) = 42,31; *p* < 0.001), where male half-marathon runners presented the lowest mean age (Table 1).

### 3.1. Age x Continent of Athletes’ National Citizenship

**Figure 1:**
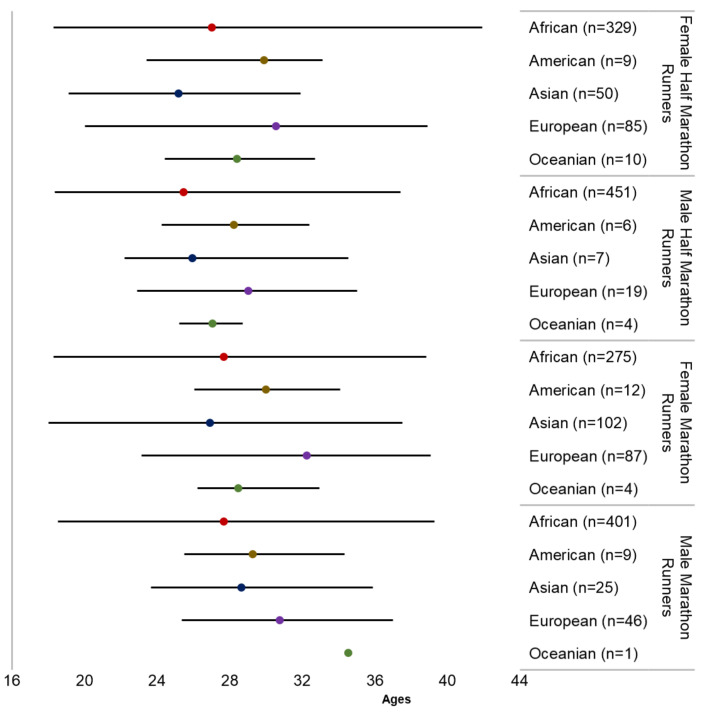
Runners mean age and age range (min–max), by sex and race distance, stratified by continent.

The runners were from the five continents (Africa, 1456 runners—75.4%; Europe, 237 runners—12.3%; Asia, 184 runners—9.5%; America, 364 runners—1.9%; Oceania, 19 runners—1.0%). The analysis of variance indicated a significant difference in the mean age of peak performance between the continent of athletes’ national citizenship (F (4, 1884) = 62.85,601; *p* < 0,001), with Asian runners presenting the lowest mean age (26.6 ± 3.7 years), while their European peers presented the highest mean age (31.0 ± 3.9 years). Taking into account both sexes together, statistically significant differences were observed: African runners were younger than their American (Δ: −2.68; 95% CI = −4.4–−0.7) and European peers (Δ: −4.2; 95% CI = −5.0–−3.4), while Asian runners were younger than Americans (Δ: −2.8; 95% CI = −4.8–−0.8) and Europeans (Δ: −4.4; 95% CI = −5.5–−3.3); no statistically significant difference was observed between Oceanian runners and their peers from other continents. Figure 1 presents the runners’ mean age, as well as the age range (minimum and maximum), by sex and race distance, stratified by continents.

### 3.2. Sex x Race Distance

**Figure 2:**
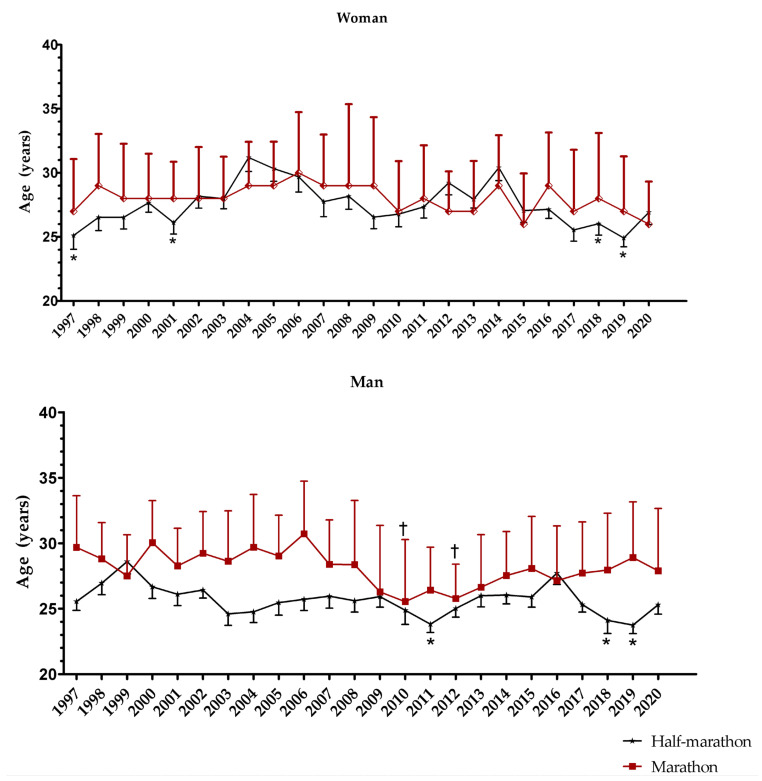
Mean age of the peak of performance, by sex and distance, between 1997 and 2020. For women, * indicates differences compared to 2004; while for men * indicates differences compared to 1999; † indicates differences against 2006.

Figure 2 presents results from the analysis of variance by sex and race distance. Among female marathoners, no statistically significant difference for age alongside the years (F = 1.15; *p* = 0.279) was observed, while among female half-marathoners, a statistically significant difference was observed, where runners in the 2004 ranking presented the highest mean age (31.1 ± 4.8 years) when compared to their peers in the years 1997 (25.1 ± 4.7 years; Δ = 6.0; 95% CI = 1.0–11.1), 2001 (26.1 ± 3.8 years; Δ = 5.0; 95% CI = 0.05–10.12), 2018 (26.0 ± 4.8 years; Δ = 5.1; 95% CI = 0.19–10.13), and 2019 (24.9 ± 3.9 years; Δ = 6.2; 95% CI = 1.24–11.31). Among males, statistically significant differences were observed in both distances: among half-marathoners, those in the rank in 1999 presented the highest mean age (28.6 ± 4.05 years), when compared against runners in the 2011 (23.8 ± 2.8 years; Δ = 4.7; 95% CI = 0.41–9.15), 2018 (24.1 ± 4.2 years; Δ = 4.4; 95% CI = 0.01–8.98), and 2019 (23.7 ± 2.7 years; Δ = 4.8; 95% CI = 0.37–9.34) ranks; further, among marathoners, those in the 2006 rank (30.71 ± 4.03 years) showed a statistically significant higher mean age when compared to those in the 2010 (25.5 ± 4.7 years; Δ = 5.1; 95% CI = −0.21–8.83) and 2012 (25.7 ± 2.6 years; Δ = 4.9; 95% CI = 0.30–9.58) ranks.

### 3.3. Age x Ranking Position

**Figure 3:**
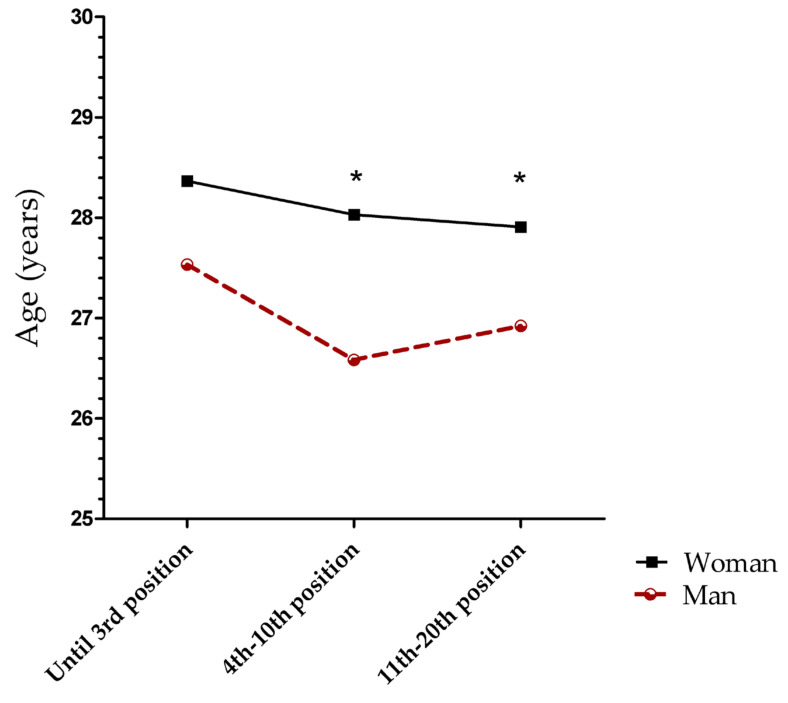
Mean age differences, taking into account runners’ groups (derived from their ranking positions), between sexes. The * indicates statistically significant differences between sexes.

When runners were classified based on their ranking position, no significant interaction between sex and their ranking was observed. However, statistically significant differences were observed between sexes (F (1, 1879) = 23,114; *p* < 0.001) for categories “4th–10th positions” (women: 28.0 ± 4.03 years; men: 26.5 ± 3.7 years) and “above the 10th position” (women: 27.8 ± 4.3 years; men: 26.9 ± 4.1 years) (Figure 3).

## 4. Discussion

The purposes of this study were to identify the possible trend in the age of peak performance in endurance athletes of marathon and half-marathon between 1997 and 2020 and to identify differences in the age of peak performance of athletes between continents. The main results indicated that: (1) no significant differences were observed in the age of peak performance among female marathoners between 1997 and 2020, and this group presented the highest mean age for peak performance; (2) taking into account the continent of athletes’ national citizenship, Asian runners presented the lowest mean age of peak performance, while their European peers presented the highest mean age; and (3) no interaction was observed between sex and their ranking position, but women were older in both ranking position groups (“4th–10th positions” and “11th–20th position”) than men.

### 4.1. Peak Performance by Distance and Sex

In general, the age of peak performance seemed not to change across the last two decades significantly. For example, when considering the mean ages per year, the lowest mean value was observed for male half-marathoners in 2019 (23.7 years), while the highest value was found for female half-marathoners in 2004 (31.1 years). Considering sex and race distance, significant differences between groups in the mean age of peak performance were observed, where male half-marathon runners presented the lowest mean age (25.6), while female marathoners showed the highest mean age (28.4). Regarding the male age values, the present study is following a systematic review conducted by Allen and Hopkins [3], where it was reported that middle distance and marathon athletes presented a mean age range between 24 and 31 years. Differences between male half-marathoners and marathoners can be related to specific physiological adaptations for each discipline. For example, a study conducted by Coso et al. [22], involving 22 experienced runners, who had to run at the same running speed, found that completing a marathon induced a higher muscle fiber damage, perceived muscle pain level and higher body water and electrolyte deficits than half-marathon finishers. Additionally, since endurance performance is mainly determined by aerobic capacity, running economy, and lactate threshold [21], which are developed by training/practice, the marathon runners may need to spend more time in deliberate training, which can lead them to reach their peak after the half-marathoners. In addition, runners tend to move up in racing distances—they start in 5 km/10 km, and with increasing age, increase the distances to half-marathon and marathon.

In the present study, female runners presented a higher mean age than the range found in the review of Allen and Hopkins [3] (between 25 and 27 years). This difference may be due to the time interval between the studies or the trend (even though not statistically significant) for an increased age among female runners. A study aiming to investigate differences in the age of elite marathon runners competing in the “World Marathon Majors Series” (Berlin, Boston, Chicago, London, New York), the International Athletic Association Federation (IAAF) World Championship, and the Olympic Games, found that female athletes were older than male athletes (29.8 ± 4.2 versus 28.9 ± 3.8) in only two of the seven marathons.

Instead of this, it was impossible to observe a clear trend across the years regarding the age of peak performance among marathon and half-marathon runners. Different patterns were observed in both disciplines, with statistically significant differences in the age of peak performance were observed among female marathoners across the years, and they also tended to reach it in older ages than males. These results were similar to those observed when investigating the peak age performance in other Olympic sports, such as swimming, rowing and ice skating [12]. In a recent study, the authors reported that finalist female marathoners, who competed in the ”New York City Marathon” between 2006 and 2016, tended to achieve their peak performance in these races about 1 ± 2 years later than their male peers [5], with 27.3–29.4 years for men and 29.9–31.8 years for women [23].

Results found by Nikolaidis et al. [23] can be explained by the decrease in the male-female ratio of competitors in this event, but also by the fact that an increase in women participating in younger age groups has been observed. It should be considered that the female peak age performance is not just related to physiological aspects but also to cultural, social, and sports scenarios that should favor female engagement in sports [24]. In the past, female athletes usually retired early from sports careers, due to various reasons, such as family obligations, loss of motivation, injuries, or financial problems [12], but changes in social beliefs and patterns regarding opportunities for women in the last decades have allowed female athletes to compete until late ages. With the increase in the number of race events with financial compensation and also support teams, it has become more feasible for female athletes to continue their sports careers until older ages [25]. Other aspects that may be associated with the results are the maintenance of long-term training, aerobic capacity, experience with the discipline, and economic movement, which generally increases with age [26].

### 4.2. Age of Peak Performance and Ranking Position

When general mean age and ranking position was considered, female runners were shown to be older than male runners. Similar results were previously reported in other endurance modalities and running distances, such as mountain ultramarathon running [27], ultra-marathon running [28], 100 mile ultra-marathon running [29], and ultra-cycling [30]. A previous study, with the purpose to analyze the worldwide marathon runners ranking, considering runners ranked in the top 100, top 50, top 25, top 10, and top 3, found that male African athletes represented 70% of the runners in the top 100, and this proportion was kept up to top 3. Similar results were also observed among female runners, but with a slight difference. These findings can explain results in the present study, where no statistically significant difference was observed in the age of peak performance between sexes when considered athletes in the top 3, given that, for both sexes, this group may be majority composed by African athletes, which may have similar age, regardless of the sex [31].

### 4.3. Differences in the Age Peak Performance according to the Continent of Athletes’ National Citizenship

Regarding the continents of athletes’ national citizenship during the considered years, a predominance of African runners was observed, followed by Europeans. The African runners’ phenomenon was previously investigated [14], and studies highlighted that training characteristics [32], environmental and demographic conditions [18], genetic, anthropometric and physiological factors could be related to this supremacy [15,17]. In the present study, Asian runners presented, in general, the lowest age, except for male marathoners, where the Africans were the youngest ones. Regardless of the Kenyan and Ethiopian runners’ predominance, an increase in Asian runners among the best athletes at international level has been observed by Nikolaidis et al. [33], who investigated nationality, sex, age, and performance data between 1999 and 2015. Furthermore, it was observed that for 100 km ultra-marathons, the best performance, for both women and men, was found among Japanese athletes [34]. This increment can be explained by the fact that these athletes present an early specialization in their careers, as observed among the Africans [20].

The European athletes presented the highest mean age, ranging from 29 to 32 years, for male half-marathoners and female marathoners, respectively. It is important to note that among the “Six World Marathon Majors”, two of them are allocated in the European continent (London and Berlin), which can lead to increased participation and investments in running events in these countries, allowing athletes to maintain their careers for more time [35]. Another aspect that can be highlighted is that even with the decline in performance associated with increasing age, 12.8% of the athletes were from this continent, more than the percentage observed for those from Asia, America and Oceania.

A limitation of this study is the lack of information regarding individual characteristics that would allow a better characterization of the group (e.g., physiological, anthropometric, and socioeconomic characteristics, frequency and volume training, practice time), as well as could help in the explanation of the results, especially for female athletes that presented the oldest mean ages. Besides this, we did not consider technological, social, economic, climatic and cultural changes and the possible relationship of these factors with the performance. Another point to be mentioned is the fact that we did not consider possible changes in athletes’ national citizenship across the years. In addition, since there is no worldwide suggestion to classify the top 20 athletes into groups, based on their classification, we stratified our sample based on usual awards at individual events. Moreover, the pandemic scenario in 2020, which led to the cancelation of many race events, may not have allowed some athletes to take part in official race events, which may have led to a reduction in the representativeness of some continents. On the other hand, the strength of this study is to provide information that comprises two decades, involving the best athletes of the disciplines investigated, from all continents. In general, previous studies were conducted based on a reduced temporal time interval or taking into account data from only one country [36]. We suggest that future studies consider these age changes associated with performance changes and the pacing strategies and follow youth athletes from different continents.

## 5. Conclusions

Over the last two decades, no significant difference was observed in the age of peak performance among the worldwide best half-marathoners and marathoners for both sexes, meaning that it was not possible to identify a significant trend over the years. However, a difference was observed where in general, female half-marathoners tended to be significantly older than their male peers. When considering the rank position, independent of the distance, female athletes classified above the 4th position also tend to be older than males. Furthermore, Asian endurance runners were younger than their European peers. This information can be of relevance to developing talent identification programs and also to long-term training, given that it provides an estimation of when the peak of performance in long-distance running seems to be achieved.

## Figures and Tables

**Table 1:** Descriptive information for endurance athletes, by sex and distance, between 1997 and 2020.

	Female		Male		
	N = 483	N = 487	N = 480	N = 482	Effect Size
Variables	Half-Marathon	Marathon	Half-Marathon	Marathon	*n^2^*
Age (years)	27.5 (4.4) *	28.4 (4.1) *,†	25.6 (3.6)	28.0 (3.9) *	0.06
Race time (h:min:s)	1:07:55 (03:28)	2:22:20 (03:44)	00:59:513 (00:38)	2:06:22 (01:26)	
Running pace (min/km)	03:13 (05:32)	03:22 (05:32)	02:50 (01:82)	02:59 (02:04)	

Note: * significantly different from male half-marathoners; † significantly different from female half-marathoners; h—hour; min—minutes; sec—seconds; km—kilometer.

## Data Availability

Available online: https://www.tilastopaja.eu/db/worldtop20.php (accessed on 8 November 2020).
